# Preparation of Alumina Oxo-Cluster/Cellulose Polymers and Dye Adsorption Application

**DOI:** 10.3390/ma17236023

**Published:** 2024-12-09

**Authors:** Henglong Tang, Simeng Yao, Zhu Long, Xuefei Yang, Pengxiang Si, Chang Sun, Dan Zhang

**Affiliations:** 1 College of Textile Science and Technology, Jiangnan University 1800 Lihu Avenue, Wuxi 214122, China; 6223018029@stu.jiangnan.edu.cn (H.T.); 6213011040@stu.jiangnan.edu.cn (S.Y.); longzhu@jiangnan.edu.cn (Z.L.); zhangdan@jiangnan.edu.cn (D.Z.); 2 CETIM Technological Center, 15189 Culleredo, Spain; xyang@cetim.es

**Keywords:** aluminum oxide clusters, adsorption, dye wastewater treatment, TEMPO-mediated oxidation, balsa wood, in-situ generation

## Abstract

Aluminum oxide clusters (AlOCs) possess high surface areas and customizable pore structures, making them applicable in the field of environmental remediation. However, their practical use is hindered by stability issues, aggregation tendencies, and recycling challenges. This study presents an in -situ synthesis of AlOCs on cellulose using a solvent thermal method. The resulting adsorbent’s structural and property profiles were thoroughly characterized using multiple analytical techniques. Batch adsorption experiments were performed to assess the adsorbent’s capacity and kinetics in removing selected dyes from aqueous solutions. Additionally, both real-environment simulation and regeneration experiments have been conducted to thoroughly assess the adsorbent’s reliability, stability, and practical applicability. The aim was to engineer an effective and recyclable adsorbent specifically tailored for dye-contaminated wastewater treatment.

## 1. Introduction

The application of dyes in various industries, particularly in textile dyeing and printing, has led to the widespread discharge of dye-containing effluent into the environment [1]. These dyes, known for their colorfastness and intensity, have detrimental effects on aquatic ecosystems, including disrupting photosynthesis, affecting the aesthetic quality of water bodies, and posing potential health risks to humans through the accumulation of toxic substances [2]. Dyes with azo structures have been identified to possess carcinogenic, mutagenic, and teratogenic properties, highlighting the urgency for effective treatment methods to mitigate their environmental and health impacts [3]. Traditional approaches for dye removal encompass chemical degradation, biological treatment, and membrane separation techniques [4]. While these methods have been effective to an extent, they are often limited by high operational costs, generation of secondary waste, and the inability to completely remove certain dye compounds, especially those that are resistant to biodegradation [2,5].

Conversely, adsorption has risen as a versatile and economical alternative for dye removal [6]. This method involves the use of adsorbents to selectively bind and concentrate dye molecules from aqueous solutions. The advantages of adsorption include high removal efficiency, ease of operation, and potential for adsorbent regeneration [6]. The choice of adsorbent material is critical in determining the effectiveness of the adsorption process, with materials such as activated carbon [7,8], engineered nanoparticles, including CNTs [9,10], nanoscale metal oxides [11,12], carbon quantum dots [13], nanofibers [14], and various natural adsorbents [15,16] being widely studied and applied.

Featuring an extensive surface area and robust adsorption potential, AlOCs are considered a promising strategy for containment extraction. AlOCs-based adsorbents have demonstrated encouraging outcomes within the realm of environmental remediation: Liu et al. [17] developed a heterometallic framework with AlOCs. This material effectively eliminates iodine from cyclohexane solution with an exceptionally high removal rate of 98.8% and a considerable loading capacity of 555.06 mg g^−1^. Sun et al. [18] incorporated a single lanthanide ion into an AlOCs, effectively removing anions like KI/I_2_, ReO_4_^−^, and MnO_4_^−^ from nuclear industry waste. Luo et al. [19] devised Al_8_, and a variety of pores created by PT ligands endow these materials with notable adsorption capacity toward iodine vapor molecules. The adsorption capacities are 0.73 g g^−1^ for AlOC-151 and 0.86 g g^−1^ for AlOC-155. Zhang et al. [20] produced a family of nanosized rings, spanning from 6- to 12-membered, using a one-pot method. The adsorption capacity of the obtained AlOC-61 adsorbent reached up to 732.44 mg g^−1^ for I_2_. However, AlOCs are hampered by challenges such as limited diffusion, a propensity for aggregation, and inadequate stability, significantly constraining their developmental prospects. Consequently, these limitations have underscored the urgent need for innovative solutions.

To facilitate the recycling of powdered adsorbent materials and enhance their reusability while simultaneously diminishing the mass transfer resistance during adsorption, the adsorbents were loaded onto porous substrates, including kraft pulp fibers, nano-fibrillated cellulose combinations [21], graphene oxide nanosheets [22], magnetic Fe_3_O_4_ nanoparticles [23], and copper silicate nanotubes (CuSiNT) [24]. Wood possesses immense potential as an adsorbent material, attributable to its inherent natural abundance, biodegradability, and capacity for modification to augment its adsorptive attributes [25]. Wood biowaste material has specific reactive centers—functional groups, such as aliphatic and phenolic hydroxyl, methoxy, and carbonyl groups, which can be used as a good base for chemical modification to produce highly efficient sorbents [26]. Furthermore, the intrinsic porosity of wood endows it with a labyrinthine network of channels and cavities, which are adept at effectively ensnaring and sequestering pollutants [27].

**Scheme 1:**
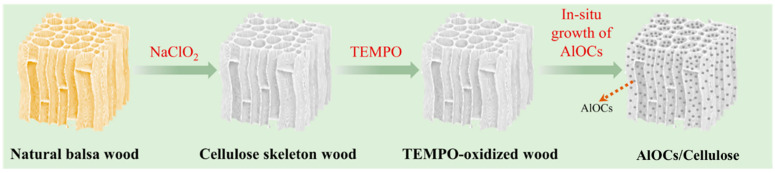
Preparation of AlOCs modified wood.

To overcome issues such as poor stability, challenging recovery processes, and aggregation tendencies, we have developed an efficient and recyclable adsorbent tailored for dye wastewater treatment. By employing a solvent thermal method, AlOCs were in -situ grown on balsa wood, thereby enhancing mass transfer, specific surface area, and recoverability (as illustrated in Scheme 1). The structure of the adsorbents is characterized using various techniques, and their dye adsorption performance is investigated in this research.

## 2. Synthesis of AlOCs/Cellulose

### 2.1. Preparation of Carboxylic Acid Functionalized Woods

Materials used for the synthesis of the adsorbent were provided in the Supporting Information. The carboxyl-functionalized cellulose scaffold was fabricated using a method that has been documented in prior research [28]. Specifically, 1 g of balsa wood was reacted with 100 mL of 1 wt% NaClO_2_ buffer solution at a pH of 4.6 and maintained at a temperature of 80 °C for a duration of 20 h. This process was conducted to eliminate lignin and hemicellulose components from wood. The sample was then thoroughly rinsed with deionized water and transferred to a solution containing 0.10 g 2,2,6,6-Tetramethyl-1-piperidinyloxy (TEMPO) and 0.75 g NaBr in 100 mL of deionized water. The oxidation process commenced with the dropwise addition of 10 mL of sodium hypochlorite solution. Concurrently, a 0.10 M sodium hydroxide solution was added to sustain the reaction mixture at a pH of 10.0. This oxidation was maintained at 25 °C and allowed to proceed for a duration of 8 h. Afterward, hydrochloric acid (0.50 M) was added to adjust the pH to neutral and terminate the reaction. The oxidized wood was thoroughly washed using deionized water to remove any residual chemicals or impurities and freeze-dried to obtain the carboxyl-functionalized cellulose skeleton.

### 2.2. Preparation of AlOCs/Cellulose

The preparation of AlOCs/Cellulose was carried out via solvothermal synthesis according to our previous research (the spatial structure of AlOCs is shown in Figure S1). Aluminum isopropoxide, benzoic acid analogs, pyrazole derivatives, and piperazine were mixed in a DMF solution at the ratio of 1:1:29.38~48.72:1 mmol (AlOC-15/Cellulose is the mixture of aluminum isopropoxide, benzoic acid, and piperazine at the ratio of 1:1:29.38 mmol; AlOC-20/Cellulose is a mixture of aluminum isopropoxide, 4-fluorobenzoic acid, and piperazine at a ratio of 1:1:29.38 mmol, and AlOC-22/Cellulose is a mixture of aluminum isopropoxide, benzoic acid, and 4-methylpyridine at a ratio of 1:1:48.72 mmol. AlOC-26-NC/Cellulose is prepared from a mixture of aluminum isopropoxide, benzoic acid, 4-methylpyrazole, and piperazine in a molar ratio of 1:1:48.72:1 mmol). The mixture was sonicated for 15 min to ensure homogeneity. Then, the uniform solution was transferred together with 1.00 g of the carboxyl-functionalized cellulose skeleton into a PTFE-lined high-pressure reactor. The solvothermal reaction was performed at 100 °C for 3 days in a convection drying oven. Upon completion of the reaction and subsequent cooling to ambient temperature, the composites underwent triple washing with DMF. They were then subjected to freeze-drying, resulting in the formation of the four adsorbents.

## 3. Results and Discussion

### 3.1. Adsorbent Characterization

Instruments used for adsorbent characterization are provided in the Supporting Information.

#### 3.1.1. SEM and EDX Analysis

**Figure 1:**
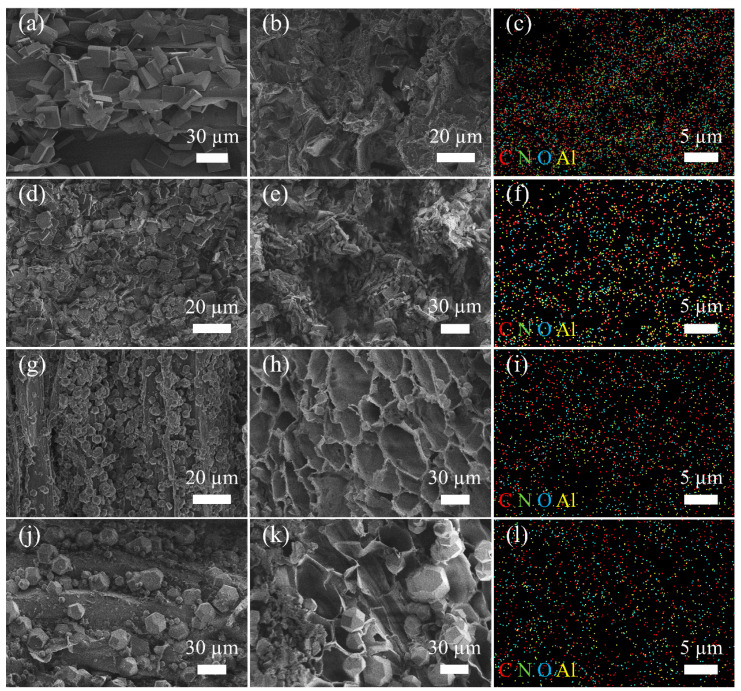
SEM images of the longitudinal sections (**a**) and cross-sections (**b**) of AlOC-15/Cellulose, as well as its EDX image (**c**); SEM images of the longitudinal sections (**d**) and cross-sections (**e**) of AlOC-20/Cellulose, along with its EDX image (**f**); SEM images of the longitudinal sections (**g**) and cross-sections (**h**) of AlOC-22/Cellulose, and its EDX image (**i**); SEM images of the longitudinal sections (**j**) and cross-sections (**k**) of AlOC-26-NC/Cellulose, as well as its EDX image (**l**).

In order to scrutinize the microstructural characteristics and the distribution of AlOCs within wood samples, Scanning Electron Microscopy (SEM) and Energy-Dispersive X-ray spectroscopy (EDX) analyses were conducted on the four prepared adsorbents. Examination of the images presented in Figure 1, specifically panels (a), (d), (g), and (j), reveals a copious deposition of AlOC-15, AlOC-20, AlOC-22, and AlOC-26-NC, respectively, along the longitudinal surfaces of the wood samples. It is discernible that AlOC-15 and AlOC-20 exhibit a regular tabular configuration, while AlOC-22 and AlOC-26-NC assume a regular polyhedral shape, corroborating the morphologies reported in the literature [29]. Cross-sectional imagery of the four adsorbents, depicted in Figure 1b,e,h,k, further demonstrates the adherence of the AlOCs to the surfaces of the wood’s cavities, with the honeycomb-like, multi-channel architecture of the wood still perceptible, thereby attesting to the successful fabrication of the AlOCs-modified wood. Additionally, the EDX images, illustrated in panels Figure 1c,f,i,l, indicate a uniform distribution of the AlOCs on the wood.

#### 3.1.2. BET Analysis

**Figure 2:**
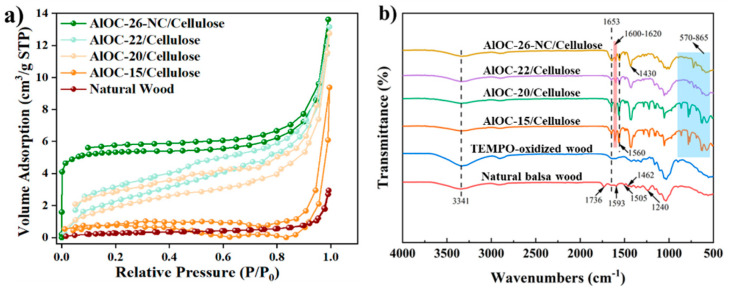
(**a**) BET analysis of the adsorbents, and (**b**) FT-IR spectra of natural balsa wood, TEMPO-oxidized wood, and the AlOCs/Cellulose.

Figure 2a and Table 1 illustrate the results of the BET analysis of the natural balsa wood and AlOC-26-NC/Cellulose. Upon functionalization with AlOC, the specific surface area of the adsorbent experienced a substantial increase, in which the specific surface area of AlOC-26-NC/Cellulose was the largest, rising from 1.06 m^2^/g to 20.66 m^2^/g, while at the same time, the pore volume increased from 0.004 cm^3^/g to 0.021 cm^3^/g, while the average pore diameter underwent a reduction, shrinking from 15.90 nm to 4.06 nm. The nitrogen adsorption–-desorption isotherms obtained for AlOC-26-NC/Cellulose exhibit characteristics of a Type II curve, which is commonly associated with materials possessing a range of pore sizes and structures. The shape of the isotherm indicates unrestricted monolayer adsorption on nonporous or macroporous adsorbents [30,31], where the adsorption process is also influenced by capillary condensation. A H3-type loop was observed, suggesting the presence of slit-shaped pores.

#### 3.1.3. FT-IR Analysis

To investigate the structural alterations of the wood samples, FT-IR analysis was conducted. Figure 2b illustrates the infrared absorption spectra of natural balsa wood, TEMPO-oxidized wood, and the AlOCs functionalized adsorbents, namely, AlOC-15/Cellulose, AlOC-20/Cellulose, AlOC-22/Cellulose, and AlOC-26-NC/Cellulose. Upon examination of the spectra of TEMPO-oxidized wood, it is evident that following TEMPO oxidation, peaks corresponding to hemicellulose at 1240 cm^−1^ and 1736 cm^−1^ vanish, concurrently with the disappearance of the lignin characteristic peaks at 1462 cm^−1^, 1505 cm^−1^, and 1593 cm^−1^, confirming the successful removal of hemicellulose and lignin from the wood cell walls [32].

The peak at 3341 cm^−1^ is indicative of the vibrational activity of hydroxyl (-OH) groups. Meanwhile, the broad peak around 1653 cm^−1^ is associated with the symmetric stretching vibration of carboxylic acid (-COOH) groups, which suggests that the hydroxyl groups at the C_6_ position have been oxidized to form carboxylic acid groups [33]. In the FTIR spectrum of AlOCs/cellulose, the strong absorption bands observed between 570 and 865 cm^−1^ are associated with the stretching vibrations of Al–-O bonds [34]. The characteristic peak at 1430 cm^−1^ is attributed to the stretching vibration of C–-N bonds, signifying the coordination involvement of pyrazole and its derivatives [35]. Additionally, the characteristic peak at 1560 cm^−1^, along with the peaks within the 1600–1620 cm^−1^ range, is indicative of the bending vibrations of the aromatic C=C bonds in the benzene ring, corroborating the presence of the benzene group [36].

#### 3.1.4. Thermal Gravimetric Analysis

**Figure 3:**
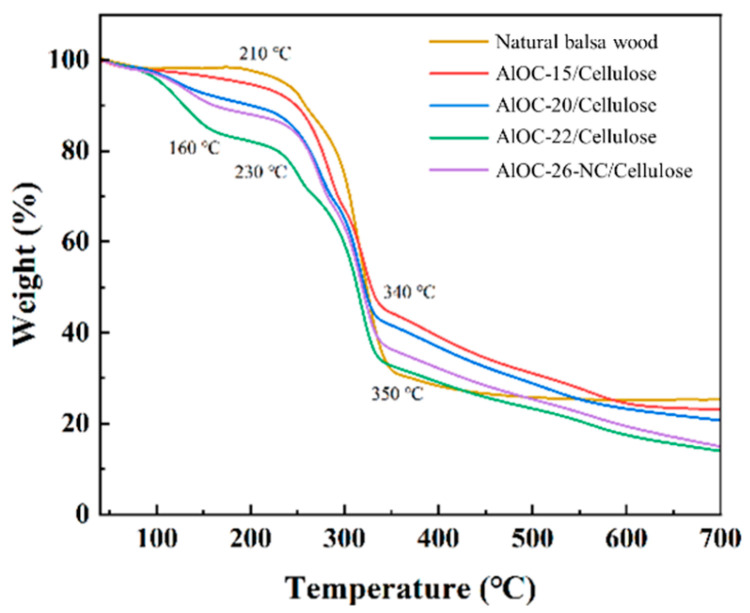
TG curves of AlOCs/Cellulose.

The thermogravimetric curves depicted in Figure 3 reveal the thermal behavior of AlOCs/Cellulose, as well as natural balsa wood. At temperatures below 160 °C, the adsorbent materials exhibited weight loss percentages of 3.96%, 8.54%, 9.85%, and 13.98% for AlOC-15/Cellulose, AlOC-20/Cellulose, AlOC-22/Cellulose, and AlOC-26-NC/Cellulose, respectively. This initial weight loss is primarily attributed to the evaporation of physically adsorbed water [37]. Natural balsa wood underwent a significant weight loss of 66.73% within the temperature range of 210 to 350 °C due to the decomposition of the wood [38]. Furthermore, the adsorbents exhibited notable weight loss behavior in the range of 230 to 340 °C, with the respective weight loss percentages being 46.92% for AlOC-15/Cellulose, 45.56% for AlOC-20/Cellulose, 46.18% for AlOC-22/Cellulose, and 48.76% for AlOC-26-NC/Cellulose, due to thermal decomposition. Between 340 and 700 °C, all adsorbents experienced a weight loss of 18 to 22%, after which the thermogravimetric curves gradually leveled off, indicating a reduction in the rate of decomposition. From these observations, it can be concluded that the incorporation of AlOCs does not significantly affect the thermal stability of the wood.

### 3.2. Adsorption Performance

Methods used to evaluate the adsorption performance were provided in the Supporting Information.

#### 3.2.1. Adsorption Selectivity

**Figure 4:**
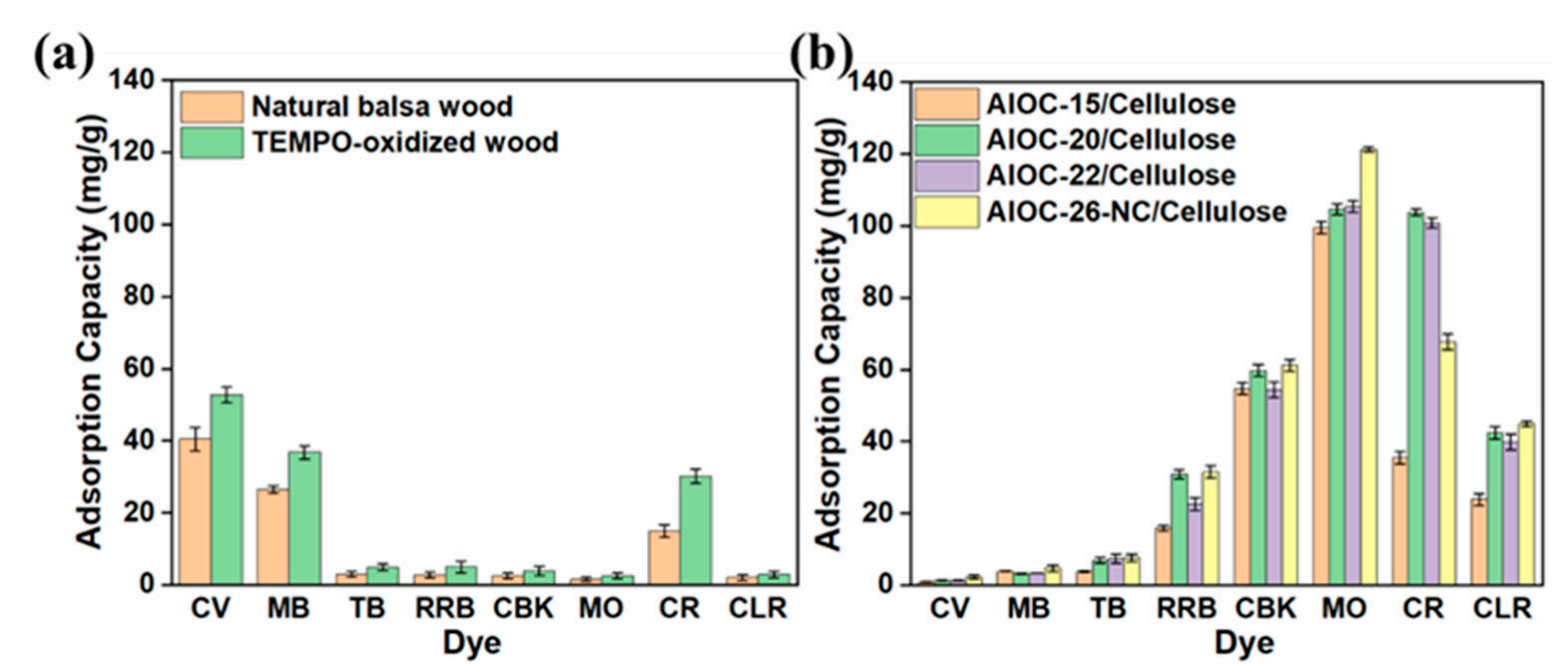
The adsorption effects of (**a**) natural balsa wood and TEMPO-oxidized wood, and (**b**) AlOCs/Cellulose (pH 7, *m* = 10 mg, CV: 204.00 mg/L, MB: 159.93 mg/L, TB: 233.30 mg/L, RRB: 239.51 mg/L, CBK: 293.20 mg/L, MO: 163.67 mg/L, CR: 348.34 mg/L, and CLR: 191.22 mg/L, 298 K, 24 h).

To explore the adsorptive capacities and selectivity characteristics of the four types of adsorbents, a set of eight organic dyes—-CV, MB, TB, RRB, CBK, MO, CR, and CLR—-were selected for adsorption experiments (the chemical structures of the dyes are shown in Figure S2). The adsorption performance of natural balsa wood and TEMPO-oxidized wood against dyes is depicted in Figure 4a. It is observed that natural balsa wood exhibits only modest adsorption for CV, MB, and MO but almost negligible adsorption capacities for the other dyes. The adsorption performance of the cellulose was enhanced after the delignification and TEMPO oxidation process. Figure 4b illustrates the adsorption performance of the four adsorbents toward various dyes. It is noted that the four AlOCs/Cellulose demonstrate analogous adsorptive characteristics for dyes with divergent molecular structures. Given that the AlOCs possess analogous structures with minor variations in the substituents of the ligands, this suggests that the substituents do not significantly influence the adsorption properties. Both the cationic dyes CV and MB are poorly adsorbed by the AlOCs/Cellulose, with negligible adsorption observed for CV and MB. This indicates that the carboxyl groups introduced by TEMPO oxidation are not the primary determinants of the adsorption performance. In contrast to the cationic dyes, the AlOCs/Cellulose exhibit superior removal efficiency for the anionic dyes (RRB, CBK, MO, CR, and CLR), with particularly notable adsorption capabilities for MO and CR. Notably, the adsorption capacity of AlOC-26-NC/Cellulose toward MO achieves 121 mg/g.

The preferential adsorption of anionic dyes by this adsorbent material is consistent with our previous findings [39]. This phenomenon can be attributed to the fact that the electron-rich region of the functional groups of the adsorbent, AlOCs, is localized on the internal walls composed of oxygen atoms, while the electron-deficient region is localized in the outer regions of AlOCs. This distribution, characterized by the exposure of the electron-deficient region to the exterior and the electron-rich region within, facilitates the preferential adsorption of anionic dyes.

#### 3.2.2. Effect of pH

**Figure 5:**
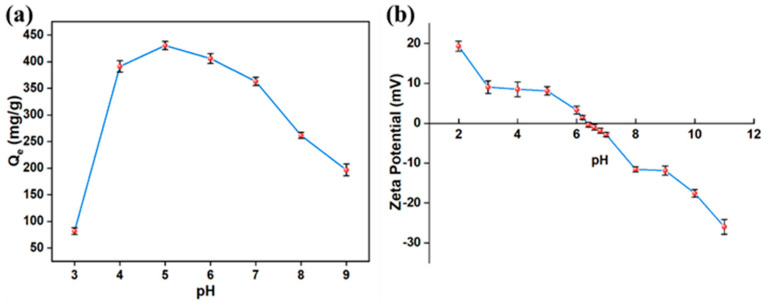
(**a**) the influence of solution pH on adsorption capacity (*m* = 10 mg, *C*_0_ = 491.00 mg/L, 298 K, 24 h); (**b**) Zeta potential of adsorbent at different pH.

The pH level of the solution is a crucial determinant of the adsorption process, as it affects the surface electrochemistry of the adsorbent and the charge status of the adsorbate. Figure 5a illustrates the adsorption behavior of Methyl Orange by AlOC-26-NC/Cellulose within a pH range from 3 to 9. At a pH of 3, the adsorption performance of the adsorbent is relatively low due to the high concentration of H^+^ ions protonated by the adsorbent, and the dye molecules lead to significant electrostatic repulsion between them. For pH values between 4 and 6, the number of positive charges carried by the dye molecules decreases, reducing electrostatic repulsion and consequently enhancing the removal capacity of the adsorbent for the anionic dye MO. Notably, at a pH of 5, AlOC-26-NC/Cellulose demonstrates the highest adsorption capacity for MO, reaching 430 mg/g. However, as the pH continues to rise, the amount of OH^-^ ions increases, which can occupy the active adsorption sites for MO. Therefore, the optimal pH for AlOC-26-NC/Cellulose to adsorb MO is determined to be 5.

The pH point of zero charge (pHpzc) represents an important characteristic of a material, describing the pH value at which the sorbent is electroneutral, i.e., it has no net charge [40,41]. As shown in Figure 5b, the zero point of charge of AlOC-26-NC/Cellulose is 6.37. Thus, the surface potential of AlOC-26-NC/Cellulose is positively charged under weakly acidic conditions. Since MO (Methyl Orange) is a sulfonic acid-based dye, it becomes negatively charged when dissolved in water. Consequently, the high adsorption capacity for anionic dyes at low pH values may be partly attributed to the electrostatic attraction between the positively charged sorbent and the negatively charged dye molecules [42].

#### 3.2.3. Adsorption Kinetics

**Figure 6:**
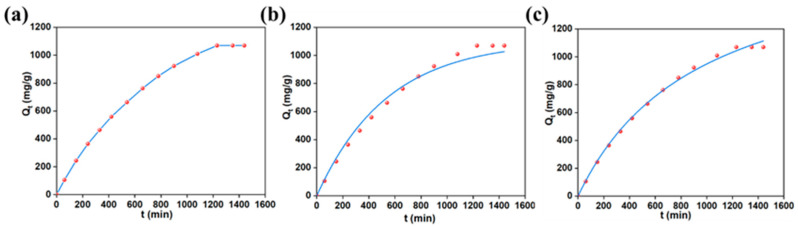
(**a**) Influence of contact time on the adsorption properties; (**b**) PFO and (**c**) PSO models for MO adsorption (pH 5, *m* = 10 mg, *C*_0_ = 3600.63 mg/L, 298 K, 24 h).

The investigation of the adsorption capacity of MO by AlOC-26-NC/Cellulose at the optimal pH (5), initial concentration (3600.63 mg/L), and temperature (25 °C) over a time range from 0 to 24 h provides insights into the adsorption kinetics. As illustrated in Figure 6a, during the initial 240 min, the slope of the curve is relatively steep, indicating a rapid adsorption rate and a significant increase in adsorption capacity. As contact time increases, the adsorption rate slows down due to the progressive occupation of the adsorbent’s active sites. When the contact time reaches 1230 min, the adsorption rate approaches zero, and the adsorption capacity stabilizes, indicating that saturation has been achieved. Consequently, the optimal contact time for AlOC-26-NC/Cellulose to MO is determined to be 1230 min.

This study of the adsorption kinetics of MO by the adsorbents, as shown in Figure 6b,c) and Table 2, indicates that the PSO model exhibits a higher determination coefficient (*R*^2^ = 0.9971) compared to the PFO model (*R*^2^ = 0.9879), suggesting that the adsorption process is primarily governed by chemical adsorption.

The kinetic fitting curves and the actual adsorption volumes were utilized to calculate the mean relative deviation (MRD) [41]. The findings reveal that the MRD for the pseudo-second-order kinetics is 0.0227, while for the pseudo-first-order kinetics, it is 0.0510. Consequently, the pseudo-second-order kinetics model aligns more closely with the observed adsorption behavior of the adsorbent.

#### 3.2.4. Adsorption Isotherms

**Figure 7:**
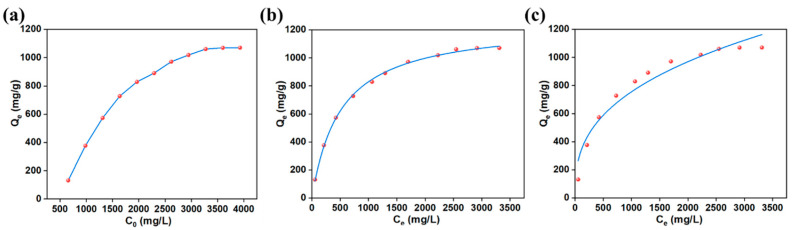
(**a**) Influences of concentration on the adsorption properties of MO; (**b**) Langmuir and (**c**) Freundlich adsorption isotherm models for MO adsorption (pH 5, *m* = 10 mg, 298 K, 1230 min).

The influence of initial dye concentration and the study of adsorption isotherms were investigated under optimal conditions of pH 5, a temperature of 25 °C, and an adsorption time of 1230 min. The adsorption effects of AlOC-26-NC/Cellulose on MO solutions within a starting concentration range of 654.66 to 3927.96 mg/L are depicted in Figure 7a. Within the concentration ranging from 654.66 to 3600.63 mg/L, the adsorption capacity for MO continually increases. As the concentration of MO increases, more active sites on the AlOC-26-NC/Cellulose are occupied by dye molecules, leading to enhanced adsorption. Conversely, at a level of 3600.63 mg/L, the capacity for adsorption levels off, which implies that the active sites have become fully occupied. At this point, the adsorption reaches saturation, with an adsorption capacity of approximately 1070.37 mg/g. Understanding the adsorption behavior of adsorbents and adsorbates at equilibrium is crucial for studying adsorption systems and determining adsorption capacities. Fitting adsorption isotherms is a reliable method for evaluating the feasibility of adsorption systems, the performance of adsorbents, and predicting adsorption mechanisms. To this end, the Langmuir and Freundlich models, both well-established in the field, were utilized to interpret the equilibrium data.

As observed in Figure 7b,c and Table 3, the Langmuir adsorption isotherm exhibits a higher determination coefficient compared to that of Freundlich. Hence, the adsorption of dye by the adsorbent is more precisely described by the Langmuir adsorption isotherm, suggesting that the adsorption process is monolayer and homogeneous.

#### 3.2.5. Study on Adsorption Properties in Simulated Real Environment

**Figure 8:**
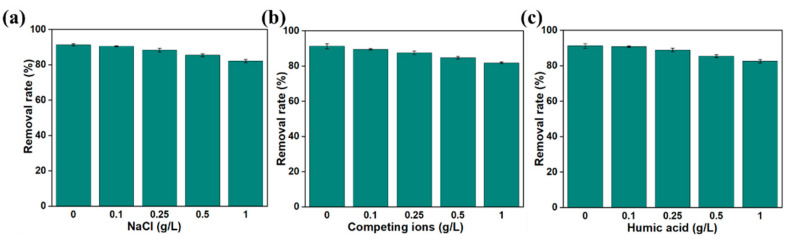
(**a**) Effect of ionic strength on MO dye removal of AlOC-26-NC/Cellulose; (**b**) Effect of competing ions on MO dye removal and (**c**) Effect of humic acid on MO dye removal (pH 5, *m* = 10 mg, *C*_0_ = 163.67 mg/L, 298 K, 24 h).

This study investigated how different interference factors impacted the adsorption of MO dye by AlOC-26-NC/Cellulose. Figure 8a illustrates the effect of ion strength by varying NaCl concentration in the MO solution. The findings indicate that as NaCl concentration increases, the dye removal rate declines. Specifically, for AlOC-26-NC/Cellulose, the removal rate was 91.26% at 0 g/L NaCl and dropped to 82.13% at 1 g/L NaCl. This trend is ascribed to two potential mechanisms: (1) increased ionic strength compresses the electrical double layer on the adsorbent’s surface, resulting in electrostatic repulsion between the adsorbent and anionic dye molecules [43]; (2) chloride ions (Cl⁻) compete with anionic dye molecules for adsorption sites, reducing the electrostatic attraction on the adsorbent surface. Their compact size (0.181 nm) gives them an edge in occupying these sites, efficiently displacing dye molecules [44].

The effects of competing anions such as Cl⁻, Br⁻, NO₃⁻, and SO₄^2^⁻ on the adsorption of MO were also examined [45], as depicted in Figure 8b. The findings aligned with the trend that dye removal efficiency decreased as the concentration of competitive ions increased. Even at an ion concentration of 1 g/L, the AlOC-26-NC/Cellulose maintained a removal rate above 80%. We also studied the influence of humic acid concentration on adsorption properties, as shown in Figure 8c. The data showed that the presence of humic acid also affected the removal rate of dyes. When the humic acid concentration is 1 g/L, the removal rate of AlOC-26-NC/Cellulose is still above 80%. The author believes that the competitive adsorption between humic acid and anionic dyes is the main reason for the decrease in dye removal rate.

#### 3.2.6. Comparative Adsorption Performance of Cellulose-Based Adsorbents

The adsorption capacities of various cellulose-based adsorbents for different dyes are comprehensively summarized in Table 4. Notably, the AlOC-26-NC/Cellulose demonstrates exceptional performance, particularly in the adsorption of MO dye. Collectively, AlOC-26-NC/Cellulose exhibits the highest adsorption capacities reported, with values of 1070.37 mg/g.

#### 3.2.7. Reusability

**Figure 9:**
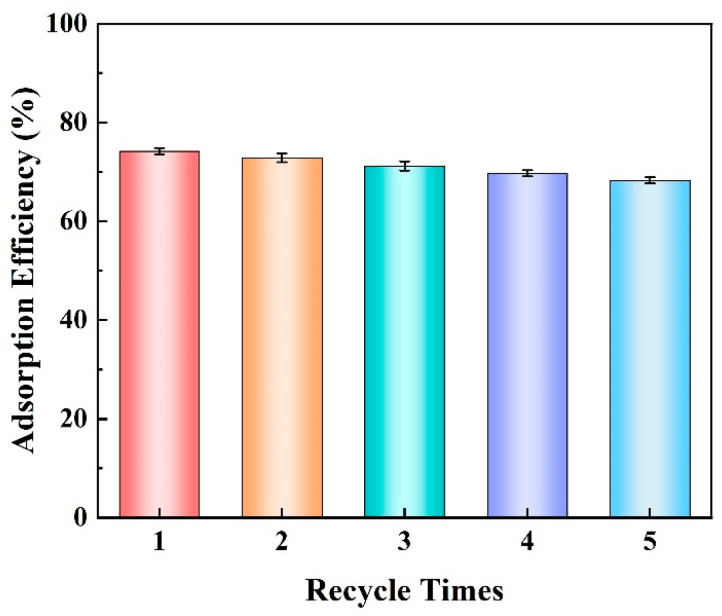
Reusability study of AlOC-26-NC/Cellulose (pH 5, *m* = 10 mg, *C*_0_ = 491.00 mg/L, 298 K, 24 h).

The regenerative capacity and reusability of an adsorbent are critical characteristics that significantly influence its practical applications. To evaluate the recyclability and reusability of the adsorbents, a series of consecutive “adsorption–-desorption” cycling adsorption experiments were conducted. Ethanol molecules containing hydroxyl groups possess polarity that enables them to form hydrogen bonds with methyl orange (MO), thereby exhibiting strong solubility. Consequently, employing ethanol as a desorption solvent is highly effective. As depicted in Figure 9, after five rounds of “adsorption–-desorption,” there is a slight decrease in the removal efficiency of AlOC-26-NC/Cellulose to 75%. This reduction in efficiency is caused by the residual dye within the adsorbent, which may clog the pores and cover some of the active adsorption sites of the adsorbent. Despite this decrease, AlOC-26-NC/Cellulose adsorbent still maintains a relatively high adsorption efficiency, indicating that it possesses good reusability and regenerative properties.

## 4. Conclusions

This study reports that the in -situ synthesis of four aluminum oxo clusters modified cellulose adsorbent, namely, AlOC-15/Cellulose, AlOC-20/Cellulose, AlOC-22/Cellulose, and AlOC-26-NC/Cellulose. The adsorbent display substantially enhanced adsorption capabilities relative to untreated balsa wood. The AlOCs/Cellulose demonstrated selective adsorption capabilities for various organic dyes. Specifically, poor adsorption was observed for cationic dyes such as CV and MB but excellent adsorption for anionic dyes, particularly methyl orange (MO), with AlOC-26-NC/Cellulose achieving a remarkable removal rate of 97%. Under optimal conditions, the adsorbent exhibited an adsorption capacity of 1070.37 mg/g. The Langmuir model and pseudo-second-order kinetics were found to align with the adsorption characteristics, indicating a monolayer and homogeneous adsorption process predominantly driven by chemisorption. The findings underscore the efficacy of AlOCs/Cellulose as adsorbents for dye removal, presenting a sustainable and efficient approach to environmental remediation and industrial wastewater management. Their superior performance, coupled with their regenerative and reusable nature, positions these adsorbents as highly attractive for practical applications and points toward a promising avenue for the advancement of innovative adsorbent materials.

## Figures and Tables

**Table 1:** Pore distribution analysis.

Adsorbent	Specific Surface Area (m^2^/g)	Pore Volume (cm^3^/g)	Average Pore Diameter (nm)
Wood	1.06	0.004	15.90
AlOC-15/Cellulose	3.05	0.014	7.34
AlOC-20/Cellulose	10.18	0.016	6.19
AlOC-22/Cellulose	18.33	0.023	3.64
AlOC-26-NC/Cellulose	20.66	0.021	4.06

**Table 2:** Kinetic model parameters.

Adsorbent	PFO			PSO		
AlOC-26-NC/Cellulose	*Q*_e_ (mg/g)	*K*_1_	*R^2^*	*Q*_e_ (mg/g)	*K*_2_	*R* ^2^
	1102.59	0.00186	0.9879	1846.25	5.724 × 10^−7^	0.9971

**Table 3:** Parameters of isothermal adsorption models.

Adsorbent	Langmuir			Freundlich		
AlOC-26-NC/Cellulose	*Q*_m_ (mg/g)	*K*_L_	*R* ^2^	n	*K*_F_	*R* ^2^
	1249.10	0.0020	0.9986	63.01	−0.3596	0.9497

**Table 4:** Dyes adsorption capacities of cellulose-based adsorbents.

Composites	Dyes	*Q*max (mg/g)	Experimental Conditions	References
MOF@wood (MIL-53(Al)@Pine-MA)	Methylene blue	54.00	pH 6.0, 48 h, 298 K	[46]
MGO@Cellulose@Lipase	Methylene blue, Malachite green	66.79, 51.87	pH 6.0, 30 min and 40 min, 298 K	[47]
Meth-Kal@Cellulose	Amido black 10B	172.14	180 min, 303 K	[48]
Graphene Oxide-ED@Cellulose	Crystal violet	260.00	pH 6.0, 80 min, 298 K	[49]
Lignocellulosic sawdust-Fe/Zn	Reactive orange 16	60.97	pH 4.0, 110 min, 298 K	[50]
Quaternary amine-modified orange peel powder	Reactive red 120 azo	344.80	pH 2.0, 110 min, 338 K	[51]
CPB-modified peanut husk	LG anionic dye	146.20	pH 2–4, 200 min, 303 k	[52]
Cationized rice husk cellulose	Congo red	580.09	pH 8, 303 K	[53]
AlOC-26-NC/Cellulose	Methyl orange	1070.37	pH 5.0, 1320 min, 298 K	This work

## Data Availability

The datasets generated during and/or analyzed during the current study are available from the corresponding author upon reasonable request.

## Supplementary Materials

The following supporting information can be downloaded at: https://www.mdpi.com/article/10.3390/ma17236023/s1, Figure S1: Spatial structure diagram of AlOCs; Figure S2: Dyes structure.
