# Supplementary material for: Preparation of Alumina Oxo-Cluster/Cellulose Polymers and Dye Adsorption Application
Source: Materials (Basel). 2024 Dec 9;17(23):6023. doi: 10.3390/ma17236023 (PMC11643062; doi:10.3390/ma17236023)
Supplement: Supplementary file 1 [file materials-17-06023-s001.zip › materials-3338579-supplementary.pdf]

Supporting Information

Preparation of Alumina Oxo-cluster/Cellulose Polymer and Dye Adsorption Application

Tang Henglong[1], Yao Simeng[1], Yang Xuefei[2], Long Zhu[1], Si Pengxiang[1]*, Zhang Dan[1]* and Sun Chang[1]*

[1]College of Textile Science and Technology, Jiangnan University 1800 Lihu Avenue Wuxi, 214122, China

[2] CETIM Technological Center, Culleredo, A Coruña-Spain

Content

Materials S2

Characterization of adsorbents S2

Adsorption performance study...S3

Effect of pH value of dye solution...S4

pH point of zero charge (pHpzc) S4

Effect of initial concentration S5

Effect of contact time...S5

Adsorption isothermal...S5

Adsorption kinetics..S6

Study on adsorption properties in simulated real environment S7

Reusability **S7**

Materials

Balsa wood was obtained from Caoxian Huajiangge Wood Industry Co., Ltd Benzoic acid, piperazine and pyrazole were obtained from Shanghai Titan Scientific Co. Ltd, 4-fluorobenzoic acid and $NaClO_2$ buffer solution were obtained from Beijing Innochem Science and Technology Co. Ltd, aluminum isopropoxide, TEMPO, sodium bromide and 4-methyl pyrazole were Shanghai Macklin Biochemical Co. Ltd. N,N-dimethylformamide, Ethanol, hydrochloric acid, sodium hydroxide were obtained from Sinopharm Chemical Reagent Co. Ltd. The eight cationic and anionic dyes, i.e., crystal violet, methyl orange, methylene blue, rose red B, thymol blue, acid chromium blue K, congo red and cresol red, were obtained from Sinopharm Chemical Reagent Co. Ltd.

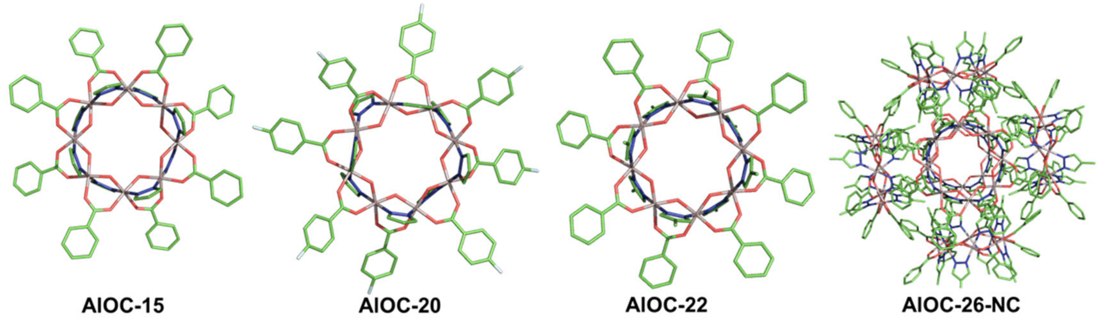

Fig.S1. Spatial structure diagram of AlOCs.

Characterization of adsorbents

The surface morphologies of the adsorbent were visualized by scanning electron microscopy (su1510, Japan). Elemental analysis was performed on an EDAX TEAM octane EDS-30 energy spectrum analyzer. Functional groups on the adsorbent surface were determined using a Fourier transform infrared spectrometer (Nicolet is10, USA)

in the range of 400~4000 cm^{-1} Thermogravimetric analysis was carried out on a fully automated thermogravimetric analyzer (METTLER TGA2) at a heating rate of 10 °C·min^{-1} from 40 °C to 700 °C. At room temperature, UV-vis spectra were recorded using UV-visible spectrophotometer (SHIMADZU, UV-2600) scanning from 200~800 nm.

Adsorption performance study

The adsorption properties of the AlOCs on eight anionic and cationic dyes viz. Crystalline Violet (CV), Methyl Orange (MO), Methylene Blue (MB), Rhodamine B (RRB), Thymol Blue (TB), Acidic Chromium Blue K (CBK), Congo Red (CR), Cresol Red (CLR) were studied. The structure of each dye is shown in Fig. S2. In the adsorption experiments, 10 mg (m) of the AlOCs were added to 10 mL of different dye solutions with an initial concentration of 0.5 mmol/L (CV: 204.00 mg/L, MB: 159.93 mg/L, TB: 233.30 mg/L, RRB: 239.51 mg/L, CBK: 293.20 mg/L, MO: 163.67 mg/L, CR: 348.34 mg/L, and CLR: 191.22 mg/L), pH=7, and shaken at a constant temperature at a uniform rate for 24 h under the temperature of 25 °C. At the end of the adsorption process, the solutions were centrifuged at 500 rpm for 5 min using a high-speed centrifuge (TG16G), and the supernatants were taken and tested using UV-vis spectrophotometer. Its absorbance was calculated from the standard curve to determine the concentration of the adsorbed dye solution. At equilibrium, the number of different dyes adsorbed per unit mass of adsorbent (Q_e) was calculated based on the following equation:

$$Q_e = \frac{(C_0 - C_e)V}{W} \quad (1)$$

Where C_0 (mg/L) is the initial concentration of the dye solution, C_e (mg/L) is the equilibrium concentration of the dye solution after adsorption, V (mL) is the volume of the dye solution, and W (mg) is the mass of the adsorbent.

The dye species and adsorbent corresponding to the maximum adsorption capacity were selected, and the effects of pH, initial concentration and adsorption time on the adsorption performance of the adsorbent were investigated in subsequent experiments to obtain the optimal adsorption conditions and the maximum adsorption capacity for the dye.

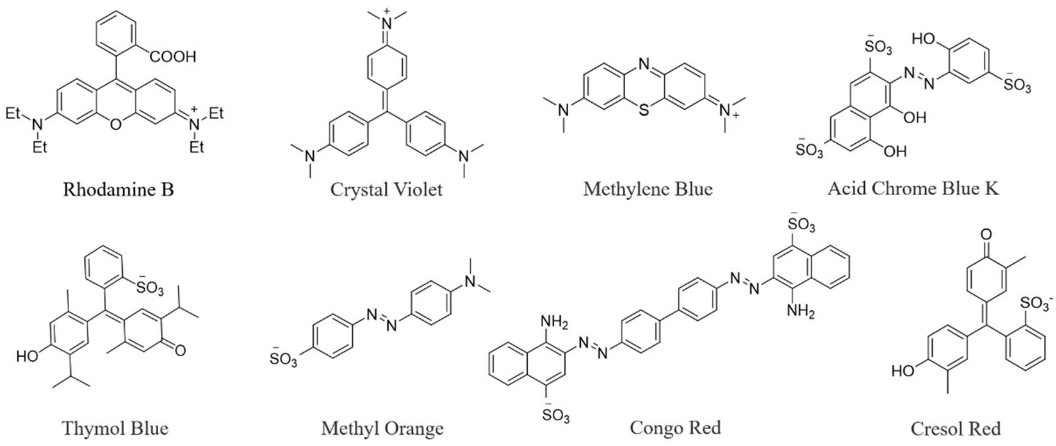

Fig.S2. Dyes structure.

Effect of pH value of dye solution

The pH of the dye solution was varied in the range of 3 to 9. In the adsorption experiments, 10 mg of the AlOCs were added to 10 mL of different dye solutions with an initial concentration of 1.5 mmol/L (491.00 mg/L), and shaken at a constant temperature at a uniform rate for 24 h under the temperature of 25 °C. The adsorption capacity at equilibrium was calculated using equation (1) to obtain the optimum pH value.

pH point of zero charge (pHpzc)

Ten milligrams of the adsorbent was weighed and placed into several 100 mL glass beakers. To each beaker, 50 mL of deionized water was added, and the mixture was sonicated for 2 hours to ensure the adsorbent was uniformly dispersed into a stable suspension. The pH of the suspensions was then adjusted to various values using 0.1 M HCl and 0.1 mM NaCl. Following this, the zeta potential of each suspension was measured, with each sample being tested three times to minimize errors. The zeta potential data, along with the corresponding pH values of the suspensions, were utilized to generate a point plot.

Effect of initial concentration

Under the condition of optimum pH, the initial concentration was varied in the range of 2 to 12 mmol/L (654.66 to 3927.96 mg/L). Other adsorption conditions were kept consistent with the previous experimental conditions. The adsorption capacity at equilibrium was calculated using equation (1) to obtain the optimum initial concentration.

Effect of contact time

Under the conditions of optimal pH as well as optimal initial concentration, the contact time was in the range of 0-24 h, and other adsorption conditions were kept consistent with the previous experimental conditions. The adsorption capacity at equilibrium was calculated using equation (1) to obtain the optimal adsorption time.

Adsorption isothermal

Langmuir isotherm and Freundlich isotherm equations were determined to show the systematic adsorption within the system by the effect of concentration changes on the adsorption capacity. The Langmuir and Freundlich isotherm equations are shown in equation (2) and equation (3):

Langmuir isotherm equation:

$$Q_e = \frac{Q_m \cdot K_L \cdot C_e}{1 + K_L \cdot C_e} \tag{2}$$

Freundlich isotherm equation:

$$Q_e = K_F \cdot {C_e}^{1/n} \tag{3}$$

Q_m (mg/g) is the maximum adsorption capacity at adsorption equilibrium, K_L is Langmuir's constant, K_F is Freundlich's constant, C_e (mg/L) is the concentration of solution at adsorption equilibrium, and Q_e (mg/g) is the maximum adsorption capacity that can be achieved by the same adsorption conditions under different concentration conditions.

Adsorption kinetics

The kinetics of the pseudo-first order and pseudo-second order were determined based on the effect of time variation on the adsorption capacity of the dye. The two model fitting equations are shown in equations (4) and (5), respectively:

The pseudo-first order kinetic model equation:

$$Q_t = Q_e \cdot (1 - e^{-K_1 \cdot t}) \tag{4}$$

The pseudo-second order kinetic model equation:

$$Q_t = \frac{{Q_e}^2 \cdot K_2 \cdot t}{1 + Q_e \cdot K_2 \cdot t} \tag{5}$$

K_1 is the rate constant for the pseudo-first order model, K_2 is the rate constant

for the pseudo-second order model, Q_e (mg/g) denotes the adsorption capacity when the adsorption equilibrium is reached, and Q_t (mg/g) denotes the adsorption capacity at the moment *t*.

The mean relative deviation (MRD) was calculated by

$$MRD(\%) = \frac{\sum_{i=1}^{n}|q_{i,exp}-q_{i,cal}|}{\overline{q_{exp}}\times n} \quad (2)$$

Where $q_{i,exp}$ (mg/g) is the experimentally obtained amount of sorbed dye at experimental point i, $q_{i,cal}$ (mg/g) is calculated amount of sorbed dye at experimental point *i* by some kinetic or isotherm model and *n* is number of experimental points.

Study on adsorption properties in simulated real environment

To investigate the impact of ionic strength, varying amounts of NaCl were introduced to a 0.5 mM (163.67 mg/L) MO dye solution, which was subsequently adsorbed by AlOC-26-NC/Cellulose. The removal rate was then determined using Equation (1).

In the studies investigating the influence of competing ions, equal weights of KCl, KBr, KNO_3, and K_2SO_4 were added to the 0.5 mM (163.67 mg/L) MO dye solution. Subsequent to this, the solutions were adsorbed by the AlOC-26-NC/Cellulose, and the dye removal rate was calculated using Equation (1).

For examining the effect of humic acid, various concentrations of humic acid were mixed with the 0.5 mM (163.67 mg/L) MO dye solution, adsorbed by the AlOC-26-NC/Cellulose, and the removal rate was calculated using Equation (1).

Reusability

In each adsorption cycle, 10 mg of the AlOC-26-NC/Cellulose adsorbent was added to a volumetric flask containing a 1.5 mM (491.00 mg/L) methyl orange solution

at pH 5 and a temperature of 298 K. The mixture was agitated at room temperature for 24 hours to achieve adsorption equilibrium, after which the adsorption capacity was measured. For the desorption process, the adsorbent was immersed in an ethanol solution and stirred for 4 hours. The adsorbent was then washed three times with 30 mL of deionized water, dried, and redeployed in the next cycle of the adsorption process.
